# Rising competition in postgraduate dental training in Türkiye: implications for healthcare workforce planning (2015–2025)

**DOI:** 10.3389/fmed.2026.1807456

**Published:** 2026-04-29

**Authors:** İpek Çubukcu Özekin

**Affiliations:** Department of Restorative Dentistry, Faculty of Dentistry, Karadeniz Technical University, Trabzon, Türkiye

**Keywords:** dental education, graduate dental education, health workforce, internship and residency, workforce planning

## Abstract

**Introduction:**

Access to postgraduate dental specialty training is shaped by national selection and placement systems as well as training capacity, and constitutes an important component of healthcare workforce planning. In Türkiye, despite the recent increase in the number of dental graduates, the relatively limited expansion of specialty training positions has raised concerns about growing competition for access to dental specialty programs. This study aimed to examine long-term trends in competition for dental specialty training based on Dental Specialty Examination (DUS) data, from a healthcare workforce perspective and in relation to the international context.

**Methods:**

This retrospective study analyzed official data from the Dental Specialty Examination (DUS), the national postgraduate selection examination for dental specialty training in Türkiye, covering the period from 2015 to 2025. Data on applicant numbers, specialty training quotas, and placement outcomes were obtained from national statistics. Competition intensity was assessed using the applicant-to-quota ratio for each examination period. To account for the transition from annual to biannual examinations after 2023, temporal trends were analyzed using multiple linear regression, including examination period and exam frequency as independent variables.

**Results:**

Across 14 examination periods, the number of applicants demonstrated a consistent upward trend, while specialty training quotas showed year-to-year fluctuations without proportional growth. The applicant-to-quota ratio increased from 4.16 in the first examination period analyzed (2015) to 10.07 in the final examination period (2025). Multiple linear regression analysis indicated a statistically significant rise in competition intensity over time (β = 0.36, *p* = 0.032), whereas examination frequency was not a significant predictor (*p* = 0.853). Orthodontics and Oral and Maxillofacial Surgery consistently showed the highest competition levels and placement score thresholds among all specialties.

**Conclusion:**

Access to dental specialty training in Türkiye has become increasingly competitive over the past decade, reflecting structural constraints within the dental workforce system rather than shifts in individual career preferences alone. The findings highlight the importance of considering national specialty examinations not only as placement mechanisms but also as indicators of healthcare workforce dynamics, underscoring the need for multidimensional and internationally informed approaches to dental workforce planning and specialty training capacity.

## 1 Introduction

Oral health is an integral component of overall health and wellbeing (1) and sustaining population health relies on having enough skilled oral healthcare professionals. The World Health Organization highlights that reaching national and global health objectives depends on developing health workers who are motivated and supported by suitable working conditions (2) According to the latest OECD indicators, the average dentist-to-population ratio across member nations is approximately 7.0 per 10,000 residents, with most European nations maintaining a ratio between 5.5 and 8.5 (3). While increasing the number of dentists is often seen as a way to improve access to oral healthcare, a rapid rise in graduate numbers that does not match public and private sector job opportunities can lead to unintended issues, such as insufficient public employment, reduced income levels, dissatisfaction among professionals, and heightened competition for postgraduate specialty training. Evidence from countries like Saudi Arabia, India, and Chile shows that expanding dental education capacity quickly has increased the number of new graduates entering the workforce, while also limiting public sector job opportunities and intensifying competition for postgraduate specialties (4–6).

In healthcare systems where public sector employment opportunities are limited and private practice predominates, postgraduate specialty training offers new graduates a more predictable career pathway. In this context, the growing demand for specialization reflects structural constraints within the employment system rather than purely academic aspirations. Türkiye represents a notable case for examining these dynamics. The relatively higher likelihood of employment in the public sector as a “specialist dentist” is considered a key determinant of specialization preferences among Turkish dental graduates. Accordingly, 52.3% of dental students have reported including specialty training in their post-graduation career plans, while only 5.9% indicated that they did not intend to pursue specialization (7). Uncertainty regarding employment opportunities in general dental practice, financial advantages associated with specialization, and the preference for working within a single discipline have been identified as major factors influencing students’ interest in specialty training (8).

Access to dental specialty training in Türkiye has been regulated through a centralized ranking examination since 2012, known as the Dental Specialty Examination (DUS). This examination is administered by the Measurement, Selection and Placement Center (ÖSYM), the national examination authority in Türkiye, and candidates are placed into specialty programs based on their examination scores and preferences.

The DUS is a multiple-choice examination covering both basic and clinical sciences, consisting of a total of 120 questions (40 basic science and 80 clinical science questions), and is administered in a single session lasting 150 min. Through the DUS, candidates are placed into specialty programs in Orthodontics, Oral and Maxillofacial Surgery, Endodontics, Periodontology, Pediatric Dentistry, Prosthodontics, Restorative Dentistry, Oral and Maxillofacial Radiology, and Oral Pathology (9). This examination system offers a valuable quantitative measure for monitoring the mismatch between rising demand for specialty training and limited training capacity.

In recent years, undergraduate dental education in Türkiye has expanded rapidly. The number of dental schools increased significantly from the early 2000’s, especially between 2010 and 2019, when 63 new dental faculties (42 public and 21 private) were established. An additional 13 faculties were added between 2020 and 2025, bringing the total to 105, with 75 public and 30 private institutions. The sharp rise in graduate numbers, along with candidates from previous examination periods, has led to a substantial increase in DUS applications. However, the limited growth in specialty training quotas has created a structural capacity and competition issue that goes beyond individual career choices, putting more pressure on early-career dentists and making the examination environment increasingly competitive.

Changes in policy have influenced how often the DUS is offered. The exam was given once a year from 2015 to 2022, then twice a year starting in 2023. In 2025, there was a plan to go back to one exam annually; however, after feedback from professional organizations and the public, it was decided that two exams would continue in 2026, with a permanent switch to one exam annually planned afterward. This changing exam schedule plays a key role in healthcare workforce policy, affecting competition for specialty training and candidates’ exam strategies.

While descriptive analyses of DUS data exist in the national literature, a comprehensive evaluation integrating long-term trends in training quotas, applicant volume, and placement outcomes from a healthcare workforce perspective—while providing international context—has yet to be presented. Therefore, the aim of this study is to assess the escalating competition for dental specialty training in Türkiye by analyzing DUS metrics between 2015 and 2025. To provide a structured assessment, the following research questions were addressed:

RQ1: How has the relationship between applicant numbers and available quotas in the Dental Specialty Examination (DUS) evolved between 2015 and 2025, and how is this trend distributed across different dental specialties?

RQ2: What are the main factors underlying the observed increase in demand for specialty training, and what are the potential implications of this trend for candidates and the healthcare system?

RQ3: Is the surge in demand for dental specialty training observed in other international contexts, and how is this phenomenon managed across different countries?

RQ4: Based on the findings, what evidence-based policy interventions and solutions can be proposed to harmonize dental workforce planning and specialty training capacity in Türkiye?

## 2 Methodology

### 2.1 Study design and data sources

This study was conducted as a descriptive retrospective analysis of official data from the Dental Specialty Examination (DUS), the national postgraduate selection exam for dental specialty training in Türkiye. Since the study relied solely on publicly available aggregate data that did not include any personal or identifiable information, ethics committee approval was not necessary.

Data from all examination periods conducted between 2015 and 2025 (*n* = 14) were collected from official examination guides and statistical reports published on the national examination authority’s website. Specifically, the data were obtained from the official booklets titled “Numerical Information on Placement Results” and “Minimum and Maximum Placement Scores,” published by the Measurement, Selection and Placement Center (ÖSYM^1^). The data included specialty-specific training quotas, total training quotas, the number of applicants, and minimum and maximum placement scores for each specialty. All retrieved data were carefully extracted from these official sources and systematically organized into digital tables for analysis.

### 2.2 Specialties included

Quotas for the eight main dental specialties included in the DUS—Oral and Maxillofacial Surgery, Oral and Maxillofacial Radiology, Pediatric Dentistry, Endodontics, Orthodontics, Periodontology, Prosthodontics, and Restorative Dentistry—were evaluated separately and compared across years. Additionally, Oral Pathology, for which specialty training positions were first introduced in 2023, was also included in the analyses.

### 2.3 Data processing and outcome measures

Data processing and calculations of year-to-year changes were conducted using Microsoft Excel. For each examination period, the total number of training positions, the total number of applicants, and the applicant-to-quota ratio were determined. The applicant-to-quota ratio served as an indicator of competition intensity. To demonstrate specialty-specific competition patterns, annual percentage changes in competition ratios were also computed.

## 3 Results

Table 1 shows the training quota data for nine dental specialties—Oral and Maxillofacial Surgery, Oral and Maxillofacial Radiology, Pediatric Dentistry, Endodontics, Orthodontics, Periodontology, Prosthodontics, Restorative Dentistry, and Oral Pathology—across 14 examination periods over the 10-year study period.

**TABLE 1:** DUS training quotas (2015–2025).

Examination period	OMS	OMR	Pediatric Dentistry	Endodontics	Orthodontics	Periodontology	Prosthodontics	Restorative Dentistry	Oral Pathology	Total
2015	77	35	83	69	58	66	85	82	0	555
2016	89	38	91	84	77	84	96	97	0	656
2017	79	43	77	103	70	94	107	66	0	639
2018	110	44	121	110	115	107	109	99	0	815
2019	96	36	94	86	89	57	84	77	0	619
2020	110	108	121	106	109	108	109	93	0	864
2021	88	44	120	110	83	105	110	94	0	754
2022	133	55	125	163	92	124	213	113	0	1018
2023-1	96	22	100	98	78	76	99	55	0	624
2023-2	99	22	100	98	93	77	99	51	2	641
2024-1	98	120	92	116	68	96	121	100	1	812
2024-2	111	30	123	112	102	112	112	92	0	794
2025-1	140	14	179	126	145	128	84	17	0	833
2025-2	130	32	173	123	138	122	87	42	1	848

OMS, Oral and Maxillofacial Surgery; OMR, Oral and Maxillofacial Radiology; DUS, Dental Specialty Examination.

Across the 14 Dental Specialty Examination periods conducted between 2015 and 2025, overall training quotas showed both increases and year-to-year fluctuations. Quotas were predominantly concentrated in Orthodontics, Oral and Maxillofacial Surgery, and Pediatric Dentistry, whereas more limited training positions were available in Restorative Dentistry and Oral and Maxillofacial Radiology. Among all specialties, Pediatric Dentistry exhibited the highest average number of training positions per examination period (114.2 positions per year). In contrast, Oral Pathology, for which a total of four training positions have been allocated since 2023, demonstrated the lowest average number of positions per examination period, with a mean of 0.2 positions per examination.

**FIGURE 1:**
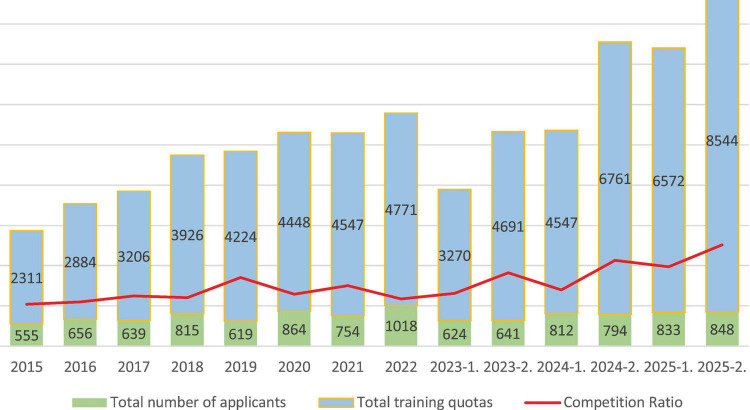
Changes in applicant numbers, training quotas, and competition ratio in the Dental Specialty Examination (2015–2025).

Figure 1 illustrates the number of applicants, total training quotas, and the applicant-to-quota ratio (competition ratio) across examination periods between 2015 and 2025.

When competition ratios, calculated as the number of applicants divided by total training quotas, were examined across examination periods, an overall increasing pattern was observed. To account for the change in examination frequency after 2023, a multiple linear regression model including both the examination period and exam frequency was applied. The analysis demonstrated that the competition ratio increased significantly over time (β = 0.36, *p* = 0.032), while exam frequency was not a statistically significant independent predictor (β = −0.23, *p* = 0.853, 95% CI: [0.037,0.686]). The overall model was statistically significant (R^2^ = 0.646, F = 10.05, *p* = 0.003), indicating a robust upward trend in competition independent of changes in examination frequency. The competition ratio showed a marked increase to 6.82 in 2019, fluctuated between 4.68 and 6.03 during the period from 2020 to 2022, and was recorded as 5.24 in the first examination period of 2023 and 7.32 in the second period. In 2024, the competition ratio was calculated as 5.60 in the first examination period and 8.52 in the second period. In 2025, the competition ratio further increased to 7.89 in the first examination period and reached 10.07 in the second examination period.

**FIGURE 2:**
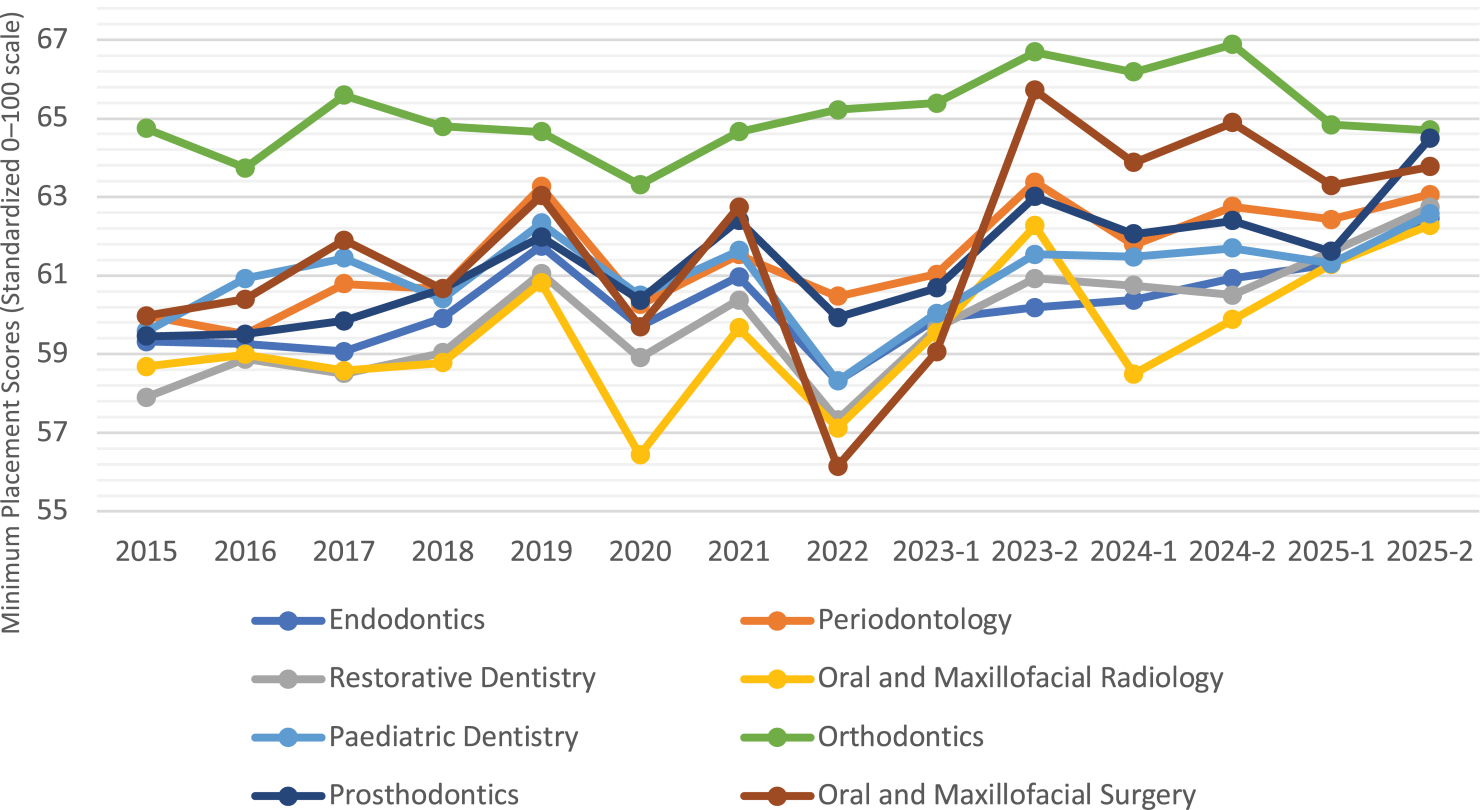
Changes in minimum placement scores by specialty (2015–2025). Scores are based on a 100-point standardized scale. The Y-axis is restricted to the 55–70 range to emphasize comparative trends and enhance visual clarity, as all data points fall within this interval.

For a total of 14 Dental Specialty Examination periods conducted between 2015 and 2025, specialty-specific minimum (cut-off) and maximum placement scores based on overall training quotas were evaluated. The results indicated that placement scores across all specialties generally exhibited an upward trend over time, with a narrowing of score ranges (difference between maximum and minimum scores) observed in some specialties. Orthodontics consistently emerged as the specialty with the highest score range across all examination periods, while Oral and Maxillofacial Surgery ranked among the specialties with the highest competition following Orthodontics. Pediatric Dentistry demonstrated fluctuations across periods but generally remained within the upper-middle score range. Endodontics was predominantly positioned within the middle score range. Although Restorative Dentistry exhibited relatively lower minimum placement scores in most examination periods, it showed a steady upward trend over time. Oral and Maxillofacial Radiology was among the specialties with the lowest minimum placement scores in certain periods (Figure 2).

## 4 Discussion

The analysis of dental specialty examination data shows a significant rise in competition for specialty training over the past decade. The findings imply that this increase is not just due to shifts in individual career goals but is also linked to the rapid growth in undergraduate dental education without a matching increase in specialty training capacity. The sharp rise in competition ratios, especially after 2023, indicates that access to dental specialty training has become more selective. The upward trend of 0.36 units identified in our findings indicates that for approximately every three examination periods, the number of candidates competing for a single specialty position increases by roughly one person (e.g., the ratio rising from 5:1 to 6:1).

In a survey of students across all class levels (years 1–5) of a dental faculty, 79.71% reported that specializing in dentistry was necessary (10). In another study of dentists currently enrolled in specialty or doctoral programs, prestige, professional title, and opportunities for academic advancement were identified as the main motivations for applying to these programs (7). Moreover, the majority of participants regarded postgraduate specialty training as an integral component of their professional careers. Previous research has also reported that a substantial proportion of final-year students and newly graduated dentists do not feel sufficiently prepared or confident to independently manage clinical practice after graduation (11). This perception may represent an additional factor driving early-career dentists toward specialization.

An examination of applicant numbers across examination periods revealed that the COVID-19 pandemic did not result in a marked change in application patterns during the 2020 and subsequent examination periods. This finding is consistent with survey-based evidence indicating that, despite high awareness of occupational infectious diseases among students planning specialty training, fear of infection did not constitute a decisive factor influencing their intention to pursue postgraduate education (12). Data from the present study further demonstrate that competition for the Dental Specialty Examination (DUS) has increased substantially over the past 3 years, reaching a level in which more than ten candidates competed for a single specialty training position in the second examination period of 2025.

Previous studies have reported a statistically significant association between gender and intended specialty preference, with male dentists more frequently selecting Oral and Maxillofacial Surgery and female dentists more commonly preferring Orthodontics as their first choice (10). In the present study, these two specialties consistently exhibited the highest minimum and maximum placement scores, as well as the highest competition ratios throughout the 2015–2025 period, suggesting that individual preference patterns are reflected in examination outcomes. This finding indicates that gender-related differences in specialty preferences may be associated with increasing competition and rising placement scores, particularly in highly demanded fields. When evaluated by specialty, professional burnout has been reported to be highest in Endodontics and lowest in Orthodontics (13). This observation suggests that specialty preferences may be closely linked to graduates’ economic expectations and that decisions to specialize are influenced not only by academic achievement, but also by perceptions of work–life balance and long-term professional sustainability. The literature further indicates that students tend to prioritize fields in which they feel more academically successful during undergraduate education (7) and that clinical internship experiences and the motivational role of faculty members play a decisive role in shaping specialty preferences (14–16).

International evidence suggests that the trends observed in Türkiye are not isolated, although policy responses to these pressures vary considerably across countries. In India, rapid growth in graduate numbers without a corresponding expansion of postgraduate training capacity has led to intense competition and access challenges (17), In contrast, in the United States, graduates’ orientation toward structured training and employment environments has been supported by the expansion of postgraduate education capacity (18, 19). Within the European context, newly graduated dentists are often described as “safe beginners,” highlighting the need for supportive transition mechanisms during the post-graduation period (20). Substantial inequalities in access to specialty training exist across European countries, and these disparities have been linked not only to national policies but also to regional socioeconomic factors (21). The fact that newly graduated dentists do not feel fully competent in clinical and administrative aspects during the post-graduation period—similar to the concept of “safe beginners” defined in Europe—leads them to perceive specialization not only as an academic goal but also as a “prestige and economic refuge.” Breaking this tendency to “seek refuge in specialization” may represent a potential strategy to reduce the excessive competitive pressure on the Dental Specialty Examination (DUS). In this context, courses focused on Dental Practice Management and Entrepreneurship, along with sector-oriented seminars, should be integrated into the undergraduate curriculum. Incorporating topics such as the legal and financial frameworks of establishing a private practice, financial management, effective patient communication, and ethical marketing into education may help eliminate newly graduated dentists’ biases toward general dental practice.

Increasing competition for access to specialty training may result in uncertainty, psychological stress, and delays in career planning for early-career dentists. Previous studies have reported associations between highly competitive environments and burnout, reduced motivation, and professional dissatisfaction (22, 23). In addition, the repeated need to prepare for specialty examinations may delay the acquisition of clinical experience and result in productive working years being devoted primarily to examination preparation. This situation may negatively affect career satisfaction at the individual level while also limiting the efficient utilization of human resources at the system level.

International experiences show that administrative and structural changes in high-stakes national specialty exams affect not only candidates’ test performance but also their professional planning, application strategies, psychosocial burden, and perceptions of fairness. In the United States, switching the USMLE Step 1 exam to a pass/fail system aimed to lower student stress; however, competition was not eliminated but simply shifted to other evaluation methods (24). In India, criticism of perceived inequities related to the two-session format of the NEET-PG exam led to a return to a single-session approach, which was seen as an effort to improve fairness among candidates. However, these structural changes have also created new logistical and planning challenges related to exam organization, preparation demands, and career timing. Likewise, the decision to revert the Dental Specialty Examination (DUS) in Türkiye from being held twice a year back to once annually may cause delays in specialization plans, longer recovery periods after exam failures, and greater career uncertainty for candidates.

**FIGURE 3:**
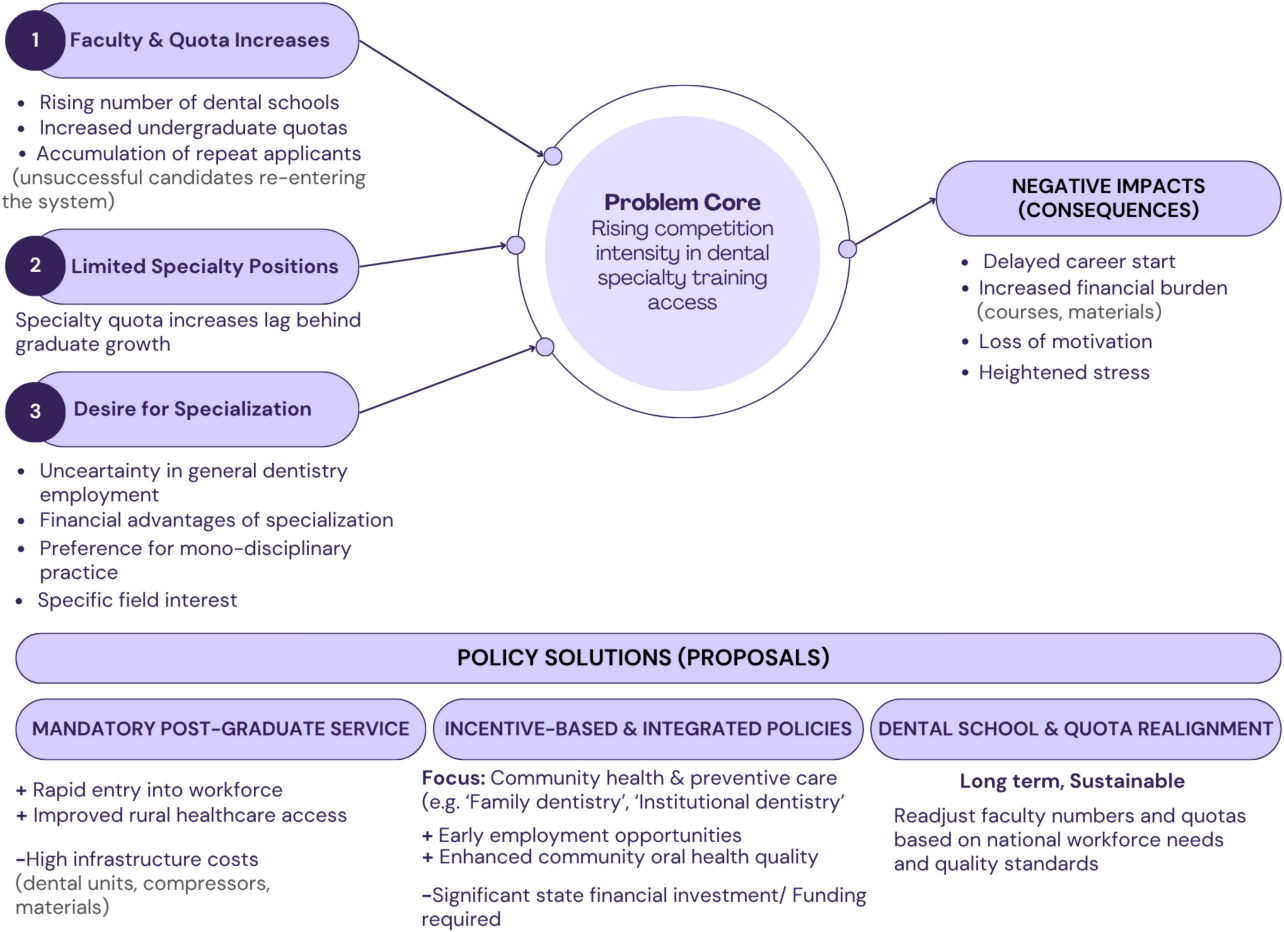
A conceptual framework for understanding the dynamics of competition in dental specialty training in Türkiye: an overview of contributing factors, systemic impacts, and potential policy options.

Figure 3 summarizes the main drivers, consequences, and potential policy responses related to competition in the Dental Specialty Examination (DUS). These findings suggest that targeted policy approaches are needed to address challenges in dental employment and access to specialty training. The mandatory service requirements implemented for medical school graduates in Türkiye after graduation is an established model that facilitates doctors’ rapid transition into professional life while also easing access to healthcare services in rural areas. Adapting this approach to dentistry could accelerate the participation of newly graduated dentists in the workforce and provide a social advantage by improving the quality of oral health services in regions with limited resources. However, while a standard examination room offers an adequate workspace for medical doctors, the need for high-cost infrastructure required for dental services—such as dental units, compressors, and specific dental consumables—constitutes a significant constraint in the widespread implementation of this model.

On a global scale, the most concrete example of this model is observed in Thailand. To prevent the uneven distribution of dentists and the “internal brain drain” from the public to the private sector, Thailand extended the mandatory service applied to medical doctors to include dentists. In this system, new graduates are obligated to work in public hospitals for at least 3 years, and there is a financial compensation fee for leaving the program (25). Although this thirty-year experience has increased the presence of dentists in rural areas, it has been emphasized that mandatory participation may create a lack of motivation and a sense of burnout among practitioners.

As an alternative to mandatory service, the incentive-based and integrative policies implemented by Brazil can be examined. Brazil integrated dentists into primary health care through the “Programa Mais Médicos” (More Doctors Program) and particularly the “Brasil Sorridente” (Smiling Brazil) national oral health policy launched in 2004. This policy increased the number of dentists in the public sector by 50% through financial investment and the creation of an extensive care network, establishing a link with public service for approximately one-quarter of the country’s dentists (26, 27). Similar to this model, structures such as “family dentistry” or “institutional dentistry” could be implemented in Türkiye; by prioritizing preventive applications and a social service-oriented approach rather than advanced treatments, both early employment and the quality of community oral and dental health could be improved.

In conclusion, although both mandatory service and incentive-based models offer alternative solutions to alleviate employment pressure, the long-term and permanent approach to solving the fundamental problem in Türkiye is the reorganization of the number of dental faculties and their quotas in line with the country’s actual workforce needs and educational quality standards.

A primary limitation of this study is that specialty training quotas are largely determined by administrative and political decisions, making predictive or causal modeling methodologically unsuitable. Consequently, the analysis was restricted to a descriptive approach focused on identifying temporal trends. Additionally, since national datasets do not differentiate between first-time and repeat applicants, the total number of candidates includes both new graduates and those from previous periods, potentially leading to an overestimation of unique individuals. Lastly, while Oral Pathology was included to ensure a comprehensive overview, its recent introduction in 2023 and the subsequent paucity of training positions (*n* = 4) necessitate caution; these sparse data may not yet reliably reflect long-term competition dynamics as established by other specialties.

## 5 Conclusion

This study demonstrates that competition for access to dental specialty training in Türkiye has increased markedly in recent years. This increase appears to be closely associated with the rapid expansion of undergraduate dental education without a corresponding increase in specialty training quotas. Consequently, access to postgraduate dental education has become increasingly selective.

These findings suggest that the Dental Specialty Examination (DUS) should be considered not merely as a placement mechanism, but as a system reflecting the dynamics of dental workforce planning, warranting multidimensional and systematic examination within the international literature.

## Data Availability

The datasets presented in this study can be found in online repositories. The names of the repository/repositories and accession number(s) can be found in the article/supplementary material.

## Footnotes

1
https://www.osym.gov.tr/
